# Heterogeneous Effects of Cognitive Arousal on the Contrast Response in Human Visual Cortex

**DOI:** 10.1523/JNEUROSCI.0798-24.2025

**Published:** 2025-04-25

**Authors:** Jasmine Pan, Louis N. Vinke, Joseph T. McGuire, Sam Ling

**Affiliations:** ^1^Psychological & Brain Sciences, Boston University, Boston, Massachusetts 02215; ^2^Center for Systems Neuroscience, Boston University, Boston, Massachusetts 02215; ^3^Department of Psychiatry, Massachusetts General Hospital, Boston, Massachusetts 02114; ^4^Harvard Medical School, Boston, Massachusetts 02115; ^5^Athinoula A. Martinos Center for Biomedical Imaging, Charlestown, Massachusetts 02129

**Keywords:** arousal, BOLD, contrast response functions, fMRI, vision

## Abstract

While animal studies have found that arousal states modulate visual responses, direct evidence for effects of arousal on human vision remains limited. Here, we used fMRI to examine effects of cognitive arousal on the gain of contrast response functions (CRFs) in human visual cortex. To measure CRFs, we measured BOLD responses in early visual cortex (V1–V3) while participants (*n* = 20, 14 females and 6 males) viewed stimuli that parametrically varied in contrast. To induce different cognitive arousal states, participants solved auditory arithmetic problems categorized as either Easy (low arousal) or Hard (high arousal). We found diversity in the modulatory effects across individuals: some individuals exhibited enhanced neural response with increased arousal, whereas others exhibited the opposite effect—a decrease in response with increased arousal. The pattern of overall BOLD modulation showed within-individual stability and was correlated with the degree of arousal-driven change in pupil size. Individuals who exhibited larger increases in pupil size with the arousal manipulation tended to show greater arousal-related decreases in visuocortical responses. We speculate that the polarity of the modulatory effect by cognitive arousal may relate to individual differences in cognitive effort expended in the high-difficulty condition, with individuals reaching different points on an underlying non-monotonic function.

## Significance Statement

While animal work suggests that arousal state has a profound impact on visual processing, the effects on human vision remain less understood. Here we assessed the influence of cognitive arousal on the neural gain of visual responses in humans to better characterize the mechanisms by which arousal affects vision. Minimal modulation was observed at the group level, but closer examination revealed substantial variability in modulation across individuals, with some showing enhancement and others exhibiting a decrease in neural modulation of visual responses with high arousal. Changes in pupil size correlated with neural modulation, suggesting a nonlinear inverted U relationship between cognitive arousal and visual processing. These results provide evidence of arousal's differential impact on vision across individuals.

## Introduction

Vision is far from static. Numerous processes dynamically influence how we perceive our environment from moment to moment. Extensive research has examined the influence of attention (Carrasco, 2011), memory (Sagi, 2011), and learning (Magnussen, 2000; Pearson and Brascamp, 2008) on vision, with many of these processes found to modulate visual processing through changes in neural gain. It is surprising, however, that less is known regarding the influence that states of arousal have on visual function, particularly in humans. Arousal's influence is putatively ubiquitous, influencing our behavioral and cognitive state, and likely plays a key role in shaping visual processing. Animal electrophysiological studies have revealed profound arousal-driven modulation of the gain of visual neural responses in the lateral geniculate nucleus and primary visual cortex (Cano et al., 2006; Niell and Stryker, 2010; Zhuang et al., 2014; McGinley et al., 2015; Vinck et al., 2015; Shimaoka et al., 2018). However, in the case of humans, only a handful of psychophysical studies have explored the relationship between arousal and the gain of visual responses, finding evidence suggestive of enhancements in contrast perception with increases in arousal (Phelps et al., 2006; Lee et al., 2014; Kim et al., 2017).

Here, we aim to characterize the underlying neural changes by exploring how arousal states influence the gain of visual responses across human early visual cortex (V1–V3) using functional magnetic resonance imaging (fMRI). We induced different arousal states and monitored arousal state changes using an approach similar to that in animal studies, corroborated by pupillometry, as changes in pupil size have been demonstrated to be tightly linked to changes in behavioral and cortical arousal states (McGinley et al., 2015; Vinck et al., 2015; Reimer et al., 2016). This approach builds upon previous pupillometry work revealing that the pupil responds to various arousal-linked factors, including cognitive load, effort, affect, and reward, with pupils dilating in heightened arousal states (Kahneman and Beatty, 1966; Beatty, 1982; Beatty and Lucero-Wagoner, 2000; Mathôt, 2018). In this study, we manipulated the difficulty of an arithmetic task to induce different cognitive arousal states, while also incorporating pupillometry to verify arousal state changes. We define cognitive arousal as arousal state intertwined with cognitive factors like effort, stress, and task difficulty. This is built on the idea that these factors are interrelated, with arousal serving as a core concept and stress and cognitive load as potential drivers (Pan et al., 2022, 2024). This cognitive arousal manipulation draws upon substantial research consistently demonstrating the impact of arithmetic difficulty on pupil size (Hess and Polt, 1964; Bradshaw, 1967; Ahern and Beatty, 1979; Steinhauer et al., 2000; Klingner et al., 2011; Pan et al., 2022), with observed changes in pupil dilation suggesting autonomic responses driven by the locus ceruleus-norepinephrine (LC-NE) arousal system (Aston-Jones and Cohen, 2005; Joshi et al., 2016). Specifically, we manipulated high and low arousal states through an auditory arithmetic task of hard and easy difficulty and explored how cognitive arousal modulates the contrast response function (CRF)—the well-established nonlinear relationship between the contrast of a signal and its corresponding neural response (Carandini et al., 1997; Priebe and Ferster, 2012). Arousal could alter the CRF profile in various ways, potentially influencing what we see and do not see.

To measure CRFs in visual cortex under the two arousal states, observers viewed parametrically manipulated contrast stimuli using the population contrast response function (pCRF) paradigm, which captures compressive nonlinearities in blood oxygen level-dependent (BOLD) CRFs with higher fidelity (Vinke et al., 2022), while concurrently solving auditory arithmetic problems of easy and hard difficulty. At the group level, we observed minimal modulation of visuocortical CRFs by cognitive arousal. However, at the individual subject level, we discovered robust and reliable individual differences in cognitive arousal's effects. Whereas some participants exhibited an enhanced gain of neural response with increased cognitive arousal, others showed no difference, and a subset displayed the opposite effect, experiencing decreased gain of response with increasing cognitive arousal. We found that the patterns of BOLD modulation were correlated with arousal-induced changes in pupil size. These individual differences may be potentially linked to interactions with higher-order cognitive brain regions and with individuals occupying different points along a non-monotonic curve relating arousal to visuocortical activity.

## Materials and Methods

### Participants

Twenty observers (mean age, 25.6 years; SD: 4.42; range, 19–35; 14 females and 6 males) participated in this study. All participants had normal or corrected-to-normal visual acuity and were recruited from Boston University and the surrounding community. Before the study, each observer provided informed consent and completed a screening form to ensure they had no MRI contraindications. All participants received compensation for their participation, except for those who are authors. The study received approval from the Boston University Institutional Review Board and was conducted following relevant guidelines and regulations.

### Apparatus and visual stimuli

The stimuli were created and presented using MATLAB (2015b) in conjunction with Psychophysics Toolbox (Brainard, 1997; Pelli, 1997; Kleiner et al., 2007) and displayed on a gamma-corrected, rear-projection screen set up within the MRI scanner bore (ProPixx DLP LED, VPixx Technologies; refresh rate, 60 Hz; resolution, 1,024 × 768 pixels) at a viewing distance of ∼99 cm.

**Figure 1.**
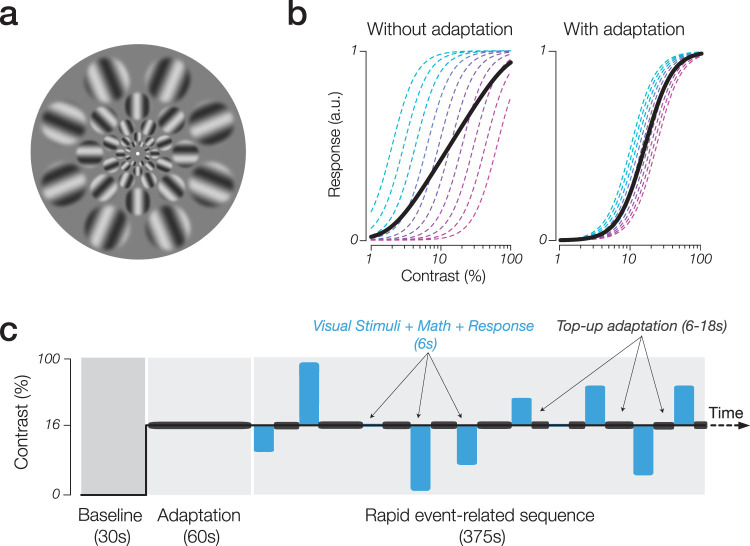
***a***, Experimental stimulus composed of gratings with radial orientations (relative to fixation) and varying spatial frequencies, which are cortically magnified. ***b***, Illustration of how sustained contrast adaptation may induce nonlinear population CRFs by bringing units within the population into closer alignment via recalibration of semisaturation point of individual CRFs. ***c***, Example timeline of an experimental run.

The visual stimuli used were adopted from Vinke et al. (2022), which were optimized to promote maximal responsiveness of the neural response within the population pCRF paradigm. The stimulus consists of an arrangement of five concentric ring patterns radiating out from fixation. Each ring is composed of eight circular apertures containing sinusoidal grating stimuli that are equally spaced, with the polar angle position of each set of apertures per ring alternating at a 22.5° offset to maximize overall stimulus spatial density across the entire visual field. Each sinusoidal grating stimulus within an aperture was set at a fixed spatial frequency and oriented radially relative to fixation, to accommodate the radial orientation bias (Sasaki et al., 2006). The spatial frequencies of the gratings in each aperture were optimized for relative spatial frequency preference following cortical magnification (Polimeni et al., 2006). Specifically, spatial frequencies of 9.38, 6.81, 4.67, 3.07, and 1.95 cycles per degree corresponded to the apertures centered at 0.9, 1.5, 2.5, 4.2, and 7° of eccentricity, logarithmically spaced out from fixation. The aperture radius increased logarithmically across successive rings from the parafovea (0.35°, innermost ring) out to the periphery (2.56°, outermost ring). To smooth the boundary between stimulus edge and mean luminance background, a Gaussian roll off (*σ* = 30) was imposed on the gratings. The inner bound of the innermost aperture ring was 0.64° from fixation, and the outer bound of the outermost aperture ring was 9.17° (Fig. 1*a*). To minimize retinal afterimages, the phase of the gratings in all apertures was randomly shifted at a rate of 10 Hz. Lastly, the luminance contrast of all the gratings across the apertures varied among nine logarithmically spaced contrast levels: 2.67, 4.0, 5.33, 8.0, 16, 32, 48, 64, and 96%.

### Arousal manipulation

To induce high and low cognitive arousal states, we employed auditory arithmetic problems categorized as either Easy (low arousal) or Hard (high arousal). Auditory arithmetic problems were used to avoid visual confounds while measuring the CRFs. Our manipulation was built upon prior pupillometry studies that utilized arithmetic problems (Hess and Polt, 1964; Bradshaw, 1967; Ahern and Beatty, 1979; Steinhauer et al., 2000; Klingner et al., 2011; Pan et al., 2022). A total of 500 unique auditory arithmetic problems were pregenerated and recorded in advance using MATLAB and Psychtoolbox's “Speak” function, featuring MacOS's “Karen” voice. In both the Easy and Hard conditions, participants were tasked with determining whether the presented arithmetic equation was true or false.

The Easy condition consisted of “add 1” equations, where participants would hear statements like “41 plus 1 equals 45” and had to indicate whether the equation was true or false. In the Hard condition, participants were presented with equations involving subtracting a number in the 10's digit. For example, the participant might hear “52 minus 18 equals 36.” In both conditions, the numbers used in any position of the equations ranged from 1 to 99, and approximately half of the equations were true, while the other half were false.

To increase the difficulty of the Hard condition arithmetic problems, we implemented several modifications to make the task more challenging. Firstly, the majority of the subtraction equations (∼78%) involved borrowing. Secondly, one-third of all incorrect answers in the session shared the same last digit as the correct answer but subtracted either 10 or 20 from the correct answer. Another one-third of the incorrect answers were centered around the correct answer, varying by ±1, 2, or 3. The remaining incorrect answers were randomly generated but always less than the first number presented in the equation. Similarly, in the Easy condition, one-third of the incorrect answers shared the same last digit as the correct answer but added either 10 or 20 to the actual answer. Another one-third of the incorrect answers varied by ±1, 2, or 3 from the correct answer. The remaining incorrect answers were randomly generated but always greater than the first number presented in the equation.

### Experimental design—population contrast response function

In our study, we utilized Vinke et al. (2022) population pCRF method to capture saturating and nonlinear population CRFs. This approach allows us to measure population CRFs using fMRI that reflect CRFs found in electrophysiological and psychophysical studies. The pCRF method is based on the concept that adaptation to a specific contrast level recalibrates neuronal population responses to the statistics of the adapter stimuli, resulting in more homogenous sensitivity across the population of neurons. This method effectively minimizes the noise that arises from averaging across a heterogeneous neuronal population with fMRI, where neurons possess varying saturation and are tuned for different sensitivities (Gardner et al., 2005; Foster and Ling, 2022; Vinke et al., 2022), the forefront explanation for linear CRFs observed in previous fMRI studies (Boynton et al., 1996; Tootell et al., 1998; Buracas et al., 2005; Buracas and Boynton, 2007; Murray, 2008; Pestilli et al., 2011; Hara et al., 2014; Itthipuripat et al., 2019).

Following the pCRF method, we utilized a fast event-related design. Visual stimuli were presented for a duration of 6 s and were interleaved with top-up adaptation periods (6–18 s) consisting of the 16% contrast stimuli. The timing of the experimental stimulus contrast presentation and top-up adaption periods was generated using the Optseq2 optimization tool (Dale, 1999). Each experimental run began with a 30 s baseline period during which participants viewed a uniform gray background with a luminance of 84.1 cd/m^2^. This was followed by a 60 s initial adaptation period, during which participants were adapted to a 16% contrast stimulus with visual properties identical to the top-up adaptation periods presented later in the event-related portion of the run. Previous studies have shown that a 60 s adaptation period is sufficient to establish a stable adapted state in the human visual system (Blakemore and Campbell, 1969). This step was taken to recenter the population response, to better capture nonlinear CRFs using fMRI.

Following the initial adaptation period, the visual stimuli were then presented concurrently with the auditory arithmetic task, during which participants had to input their response to each arithmetic problem. The simultaneous presentation of the visual stimulus and arithmetic problem, along with the response period, lasted for 6 s (see Fig. 1*a* for an illustration of the experimental run). An MR-compatible response box was used to record participants’ behavioral responses to the arithmetic task. Task trials alternated with periods of top-up adaptation that varied in duration between stimulus presentations. The top-up adaptation periods were implemented to minimize recovery from adaptation and ensure the maintenance of the initial contrast adaptation state of the visual system throughout the experimental run (Foley and Boynton, 1993; Gardner et al., 2005).

The arousal conditions, Hard (high arousal) and Easy (low arousal), were blocked by run. Runs alternated between Hard and Easy, and the first run condition, either Hard or Easy, were counterbalanced across subjects. Participants completed three to five runs of each arousal condition, each lasting 7.75 min and consisting of 465 TRs. Within each run, three trials were collected for each of the nine stimulus contrast levels. Arithmetic problems were randomized for each participant, with each participant experiencing a total of 81, 108, or 135 trials per arousal condition, depending on the total number of runs completed.

### Eye tracking and analysis

During the scanning session, we employed an MR-compatible EyeLink 1000 Plus infrared eye tracker (SR Research) to monitor the participants’ eye position. The eye tracker sampled the eye position at a rate of 1,000 Hz, and we conducted a 5-point eye calibration at the beginning of each task run. Average pupil size was obtained by converting the raw pupil data into absolute units of millimeters (Hayes and Petrov, 2016). Blinks were interpolated using cubic-spline interpolation (Mathôt, 2013). Time points with, and 1 s prior to, abnormal pupil sizes (<2 or >9 mm) were treated as signal loss and excluded from analysis. Furthermore, time points where the horizontal or vertical eye position exceeded 2.5° from the observer's mean *x* and *y* center of fixation were also excluded from analysis. Standard deviation was computed for the *x* and *y* eye positions to ensure observers were maintaining fixation. Observers who failed to maintain fixation or had excessive eye movements, with an *x* and *y* eye position standard deviation >2° and/or having >10% of the data were excluded from further eye tracking analysis. While no observers were excluded from our cutoff, we excluded one participant due to failure to collect eye data during the scan. The mean pupil diameter in millimeters was computed for each trial by averaging the pupil trace from 2 to 6 s poststimulus onset. This time window excludes the initial constriction response, in which the pupil constricts in response to changes in foveal vision (Barbur, 2004; Cherng et al., 2020).

### MRI data acquisition

The neuroimaging data were collected at the Boston University Cognitive Neuroimaging Center, on a research-dedicated 3T Siemens Prisma Scanner using a 64-channel head coil. Whole-brain anatomic data were acquired using a T1-weighted multiecho MPRAGE 3D sequence (van der Kouwe et al., 2008), using the following parameters: 1.0 mm^3^ voxels; FOV, 256 × 256 × 176 mm; flip angle (FA), 7°; TR, 2,530 ms; TE, 1.69 ms.

The functional neuroimaging data in the task runs were acquired using the following scan parameters: voxel size of 2.0 × 2.0 × 2.0 mm, 70 interleaved axial-oblique slices, a repetition time (TR) of 1,000 ms, an echo time (TE) of 30 ms, a flip angle (FA) of 64°, a field of view (FOV) of 208 mm, a simultaneous multi-slice (SMS) factor of 5, and a GRAPPA acceleration factor of 2. The SMS-EPI acquisition utilized the CMRR-MB pulse sequence developed at the University of Minnesota (Moeller et al., 2010).

### Anatomical analysis

The whole-brain T1-weighted anatomical data were analyzed using FreeSurfer's standard recon-all pipeline (Fischl, 2012). This pipeline generated cortical surface reconstructions, whole-brain segmentations, and cortical parcellations. The cortical surface reconstruction enabled surface-based registration of the functional data to the structural data, enabling alignment of the population receptive field (pRF) data to the native functional volume space for the experimental task.

### Population receptive fields

For each participant, a separate scan session was completed for pRF mapping, to delineate visual areas V1–V3. The observers underwent 3–5 scans of two distinct types of stimulus runs: (1) rotating wedge stimuli and expanding and contracting ring and (2) bar sweep stimuli. These stimuli consisted of colored objects and faces of varying spatial scale, presented on a pink noise background refreshed at a rate of 15 Hz, against a mean luminance background (Kay et al., 2013).

The collected data were analyzed using the *analyzePRF* toolbox for MATLAB, which implements the compressive spatial summation pRF (Kay et al., 2013). Only voxels located within the cortical ribbon of the occipital lobe were included in the pRF analysis. These voxels were identified using a visual area network label generated from an intrinsic functional connectivity atlas (Yeo et al., 2011), and the outcomes of the pRF analysis were utilized to manually define and draw region of interest (ROI) labels for the visual areas V1, V2, and V3.

### Functional data analysis

We utilized EPI distortion correction on all fMRI BOLD time series data. The correction was performed using a reverse phase-encode method (Andersson et al., 2003) and implemented in the functional MRI of the Brain Software Library (Smith et al., 2004). The preprocessing of fMRI data included various steps conducted with the FreeSurfer Functional Analysis Stream (Fischl et al., 2004). These steps encompassed standard motion correction procedures, Siemens slice timing correction, and boundary-based registration (Greve and Fischl, 2009) between functional and anatomical volumetric spaces.

To enable voxel-wise analyses, we did not apply volumetric spatial smoothing (FWHM = 0 mm). We employed cross-run within-modality robust rigid registration to achieve precise volumetric alignment of experimental condition data within each neuroimaging session (Reuter et al., 2010). In this process, the middle time point of the first run from each session served as the target volume, and the middle time point of each subsequent run from the same session was then used for alignment.

Before converting the BOLD time series data to units of percent signal change, we excluded time points corresponding to the initial adaptation period (60 frames). For all fMRI experimental conditions, we then conducted a univariate deconvolution analysis using a finite-impulse response (FIR) modeling approach (window size, 24 s; prestimulus delay, 4 s; Dale, 1999). This analysis yielded a set of 24 β-weight parameters describing the time course of the BOLD response for each contrast level tested.

### Voxel selection

The pRF mapping results were utilized to determine the selection of voxels within each ROI for visual cortex. These results were used to establish the boundaries of the early visual areas (V1–V3) and identify candidate voxels within each visual area that exhibited eccentricity preferences within the limits of stimulus dimensions.

To further refine the V1–3 ROI labels, voxels with poor pRF modeling goodness of fit (*r*^2^ < 20%) and unreasonably small population receptive field (RF < 0.1°) sizes were excluded. Furthermore, voxels with a maximal BOLD response exceeding 10% signal change were excluded to eliminate responses associated with draining vein hemodynamics, which are known to exhibit significant time delays compared with cortical gray matter and primarily occur at the foveal confluence (Winawer et al., 2010).

Given that the arousal manipulation involves cognitive processing, we further investigated additional ROIs beyond the visual cortex to effectively examine task- and effort-related networks and their relationship with the visuocortical responses. The network ROI labels were derived from the Schaefer-Yeo (2021) seven-network atlas with 100 cortical parcellations. We examined two main network ROIs: the dorsal attention network (DAN) and the default mode network (DMN).

### Contrast response estimation

To obtain the final contrast response estimations, we calculated the average of the FIR modeling deconvolution *β* weights within a fixed and absolute window from 5 to 10 s after the stimulus onset centered around the maximal poststimulus peak. These *β* weights were combined and averaged to generate a contrast response measurement for each of the nine contrast levels. These contrast responses were then utilized to create ROI-specific and voxel-wise CRFs for further model fitting analyses to characterize the modulatory effect of arousal on the CRFs.

### Model fitting

The resulting CRFs in each condition were then fit to a Naka–Rushton model, a descriptive model often used to capture contrast responses, as it is able to quantify the nonlinear relationship between stimulus input and response output (Naka and Rushton, 1966; Albrecht and Hamilton, 1982; Eq. 1):

$$\mathrm{Response}(c)=(R\max-b)\frac{C^{n}}{C^{n}+C_{50}^{n}}+b.\;(1)$$

**Figure 2.**
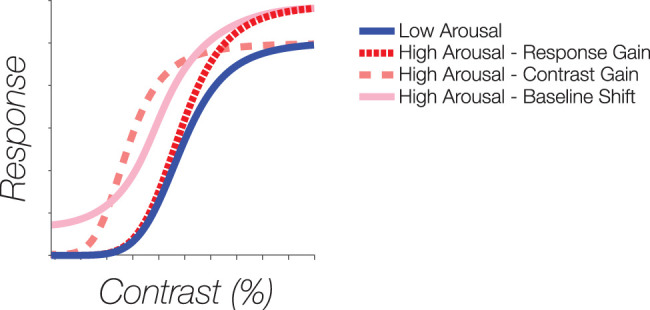
Potential modulations of the CRFs. Arousal may modulate the CRFs through (1) a response gain (red), or a multiplicative gain increasing the amplitude of the CRF; (2) a contrast gain (salmon pink), or a horizontal shift increasing sensitivity; or (3) a baseline shift (light pink), or an additive increase of the CRF.

Using a partially bounded least-squares fitting procedure in MATLAB (*fmincon*), we then assessed changes in the shape and magnitude of the CRFs between the different arousal conditions, examining key parameters quantitatively captured by the function. The parameters include the asymptote or maximum response of the CRF (*R*max), semisaturation constant (C50), slope (*n*), and baseline neural response (*b*). All of the parameters were set as free for each condition. Here, we focus on examining changes in three of the parameters: *R*max, C50, and *b*. Significant differences for any of these parameters between conditions would indicate different modulatory signatures of the CRFs by cognitive arousal. For example, one way the CRF can be altered is through a contrast gain change, or a horizontal shift of effective contrast (C50), leading to shifts in sensitivity optimized around low-to-mid contrast levels. Another way is through a response gain change, or a multiplicative gain (vertical shift in *R*max), leading to an enhanced maximum response, with the effect occurring maximally at high contrasts. Furthermore, the CRF can also be additively modulated, increasing baseline activity (*b*) for a neural population, without necessarily improving the signal-to-noise ratio for contrast perception. The upper bound of the *R*max was constrained to 10, which is considerably larger than typical observed amplitudes of the CRF. The semisaturation constant parameter (C50) was bounded between 0 and 100% contrast and the slope parameter (*n*) was bounded between 0 and 10 (see Fig. 2 for example modulations).

### Statistical analysis

For the group analyses, we employed a subject-level resampling technique to examine variation in the parameters of the fitted Naka–Rushton function between the low and high arousal conditions. We performed 100,000 bootstrap samples by randomly selecting *N*-1 subjects (sampled with replacement) from a total of *N* subjects, where *N* represents the sample size. In each bootstrap sample, we fitted Naka–Rushton functions to the average CRFs across subjects for each condition. By comparing the parameters of the Naka–Rushton function between the high arousal and low arousal conditions for each bootstrap sample, we obtained a distribution of 100,000 values representing the difference between arousal conditions. To assess the significance of these difference distributions, we calculated the 95% confidence interval as well as the proportion of values that were either greater than zero or less than zero and then doubled the smaller proportion to obtain a two-sided *p* value.

To test the reliability of the modulatory pattern by arousal on the CRFs within a given individual, a nested hypothesis test, comparing separate fits for each condition (Hard, Easy) versus one fit for both condition, was performed. For each observer, we generated 5,000 bootstrapped CRFs for each arousal condition within a visual area (V1–V3), by resampling the data across runs with replacement. Subsequently, a nested hypothesis test was performed on each of the bootstrapped CRFs, yielding an *F* statistic and corresponding *p* value. The resulting distribution of *p* values for each observer allowed us to calculate the proportion of *p* values below 0.05 across the bootstrapped CRFs. A higher proportion of *p* values <0.05 indicated that separate fits for Hard and Easy CRFs better explained the data than a single fit for both conditions—providing evidence for arousal differences on the CRF between the Easy and Hard conditions, at the level of individual subjects.

For all other analyses, Bonferroni’s correction to the number of ROIs examined was used to control for multiple comparisons.

## Results

### Increasing arithmetic difficulty hinders performance and elicits pupil dilation

In this study, we manipulated cognitive arousal through Hard and Easy arithmetic problems in order to induce, respectively, high and low arousal states. Consistent with prior literature, participants exhibited better performance in the Easy arithmetic condition (Easy mean, 98.25%; SEM: 0.69) compared with the Hard condition (Hard mean, 81.85%; SEM: 3.41; paired *t* test, *t*_(19)_ = 5.50, 95% CI [10.17, 22.64], *p* < 0.0001). This finding indicates that, on average across the group, the Hard condition was more challenging than the Easy condition. However, individual performance varied, with participants ranging from a 0% difference between Easy and Hard (achieving 99.07% accuracy in both Easy and Hard conditions) to a 50.62% difference (86.42% accuracy in Easy, 35.80% accuracy in Hard). On average, participants performed with a 16.40 ± 2.98 SEM percentage difference between the Hard and Easy conditions.

In line with previous animal studies, we utilized pupil size as a measure and proxy to differentiate between the two distinct arousal states in our subjects. Our findings replicate prior pupillometry research, demonstrating that pupil size is substantially modulated by the difficulty of arithmetic problems (Hess and Polt, 1964; Bradshaw, 1967; Ahern and Beatty, 1979; Steinhauer et al., 2000; Klingner et al., 2011; Pan et al., 2022). Specifically, we observed larger pupil size in the Hard condition (Hard mean, 4.46 mm; SEM: 0.10), compared with the Easy condition (Easy mean, 4.23 mm; SEM: 0.09). On average, there was a 0.24 ± 0.04 SEM millimeter increase in pupil size in the Hard versus Easy condition (paired *t* test, *t*_(18)_ = 6.05, 95% CI [0.15, 0.32], *p* < 0.0001). The average size of the effect was comparable with that seen in previous studies, where cognitive influences on pupil size generally range from <1–5% (Mathôt, 2018). There was variation in the magnitude of pupil modulation across subjects, with individuals exhibiting a pupil size difference ranging from −0.01 to 0.58 mm between the Hard and Easy conditions. These results provide evidence supporting the notion that subjects experienced two different cognitive arousal states driven by the arithmetic task.

### Cognitive arousal does not modulate visuocortical contrast response functions at the group level

After verifying that our cognitive arousal manipulation was effective in inducing different arousal states, we set out to address our primary question: How does cognitive arousal influence visuocortical contrast response functions? To investigate this, we measured the BOLD response in visual areas V1–V3 while we had participants solved sets of Hard versus Easy arithmetic problems (refer to Materials and Methods, Arousal manipulation) while concurrently viewing parametrically varying contrast gratings (see Materials and Methods, Apparatus and visual stimuli).

**Figure 3.**
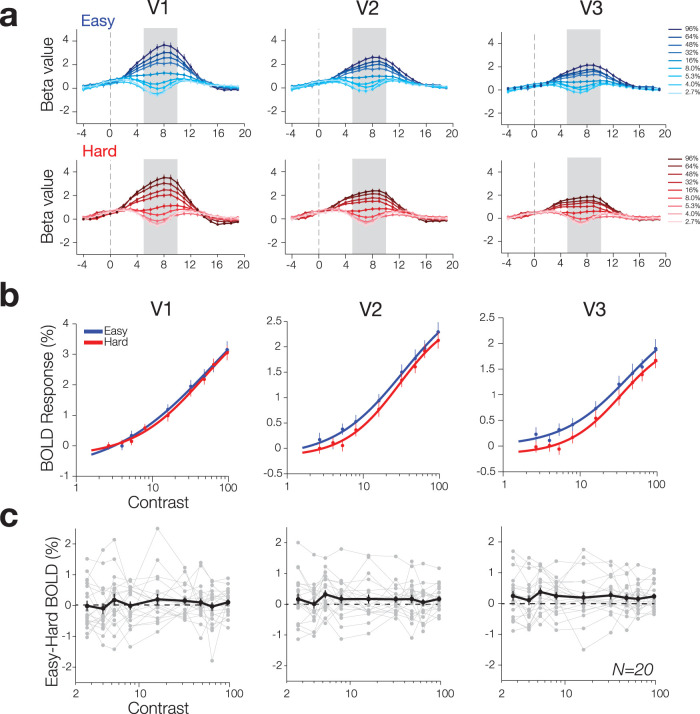
The effect of cognitive arousal on visuocortical CRFs in V1, V2, and V3. ***a***, Deconvolved HRF from the BOLD response as a function of stimulus contrast for each cognitive arousal condition, Easy (blue), Hard (red). To obtain CRFs, we measured the average response in each condition within a fixed window of 5–10 s (gray area). ***b***, CRFs for the Hard and Easy condition for each ROI. Error bars represent bootstrapped SEM across subjects. ***c***, Difference in BOLD response at each contrast level subtracting Hard from Easy. The black line represents the group average difference with the error bars representing SEM. The gray lines and dots represent the difference in BOLD CRFs for individuals, and the dashed line indicates no difference in neural response between the Hard and Easy condition. There is large heterogeneity in responses with some subjects displaying a larger CRF BOLD response in the Easy compared with Hard, others displaying a larger BOLD in Hard than Easy, and others showing little-to-no difference.

We deconvolved the hemodynamic response functions (HRFs) from the BOLD response for each cognitive arousal condition (Easy, Hard) as a function of stimulus contrast, allowing us to derive low and high cognitive arousal CRFs for each of the three visual areas (Fig. 3). To characterize the modulation of CRFs by cognitive arousal, we then fitted the CRFs in each arousal condition with the Naka–Rushton function (refer to Materials and Methods, Statistical analysis). The fitting process provided us with four essential parameters: the asymptote or maximum response of the CRF (*R*max), the semisaturation constant (C50), the slope (*n*), and the baseline neural response (*b*), which reflects the additive offset of the function from 0.

When modulated by processes such as arousal, the neural gain of the CRF profile has the potential to be altered in a variety of ways. Here we explored three modulatory effects: response gain, contrast gain, and baseline shift. Response gain refers to a shift in the *R*max parameter, signifying that cognitive arousal leads to a multiplicative gain, resulting in an increased maximal response, at the highest contrasts. Contrast gain is characterized by a shift in the C50 parameter, leading to a horizontal shift of the CRFs. Baseline shift, on the other hand, is depicted by an increase in the *b* parameter, reflecting an elevation in the baseline neural population activity.

**Figure 4.**
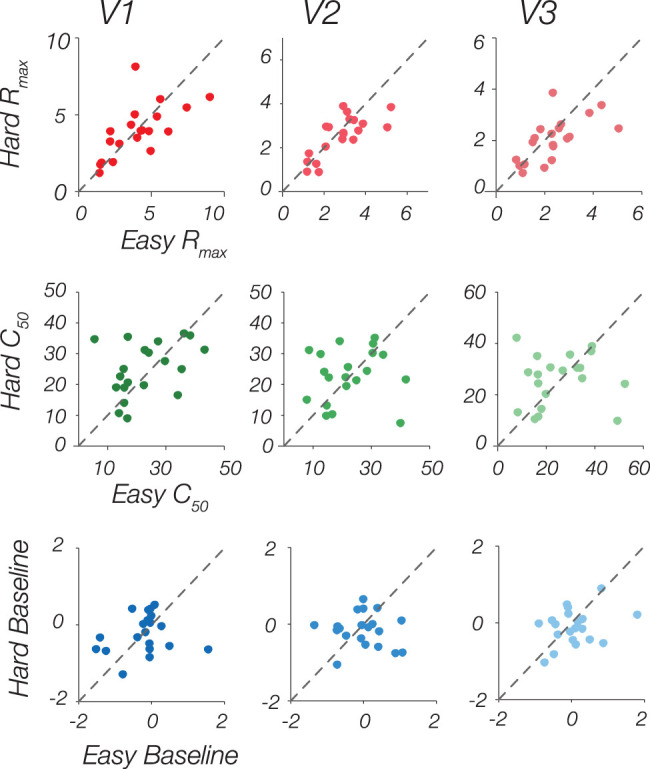
Scatterplots of Naka–Rushton parameter estimates comparing Easy versus Hard *R*max (top row), C50 (middle row), and Baseline (bottom row), for each ROI. The dashed line indicates the unity line of no difference in parameter estimates between Easy versus Hard. Overall, there is large variability in parameter estimates across participants.

While there appears to be a slight increase in neural CRF in the Easy condition compared with the Hard condition at first glance, we did not observe any statistically significant difference in overall neural response, quantified by averaging BOLD response across the CRF, between the two arousal conditions (V1: *t*_(19)_ = 1.26, *p* = 0.22, 95% CI [−0.07, 0.30]; V2: *t*_(19)_ = 1.48, *p* = 0.15, 95% CI [−0.06, 0.34]; V3: *t*_(19)_ = 1.77, *p* = 0.09, 95% CI [−0.04, 0.42]). Furthermore, there was no significant modulation by cognitive arousal observed across all three visual areas at the group level (refer to Table 1, Figs. 3, 4). The lack of significance is attributed to the variability in parameter estimates across subjects, illustrated in Figure 3*C*, which displays the difference between Easy and Hard CRFs for each observer, and in Figure 4, which displays the scatterplot of the model-fit parameters for each observer in the Hard versus Easy conditions. Notably, there are groups of subjects who exhibited a sizable increase in the parameters during the Easy condition compared with Hard, whereas others showed a sizable increase in the Hard condition compared with Easy.

**Table 1.** Naka–Rushton parameter estimates and bootstrapping results obtained from the ROI analysis

ROI		*R*max	C50	Baseline
V1	Hard (mean, 95% CI)	7.95 [5.88, 9.91]	19.87 [12.00, 26.73]	−1.12 [−2.09, −0.66]
	Easy (mean, 95% CI)	7.92 [5.51, 9.99]	15.60 [9.83, 20.71]	−1.28 [−1.89, −0.59]
	*p* value	*p* = 0.44	*p* = 0.82	*p* = 0.66
V2	Hard (mean, 95% CI)	4.01 [2.99, 6.24]	18.36 [13.40, 23.00]	−0.74 [−1.07, −0.52]
	Easy (mean, 95% CI)	4.19 [3.19, 6.24]	18.15 [12.90, 22.69]	−0.40 [−0.98, 0.04]
	*p* value	*p* = 0.40	*p* = 0.49	*p* = 0.15
V3	Hard (mean, 95% CI)	3.51 [2.36, 5.02]	22.00 [15.97, 27.50]	−0.57 [−0.86, −0.30]
	Easy (mean, 95% CI)	3.78 [2.71, 5.03]	21.43 [15.22, 29.57]	−0.17 [−0.63, 0.23]
	*p* value	*p* = 0.30	*p* = 0.38	*p* = 0.06

### Heterogeneity in arousal’s modulation across individuals

**Figure 5.**
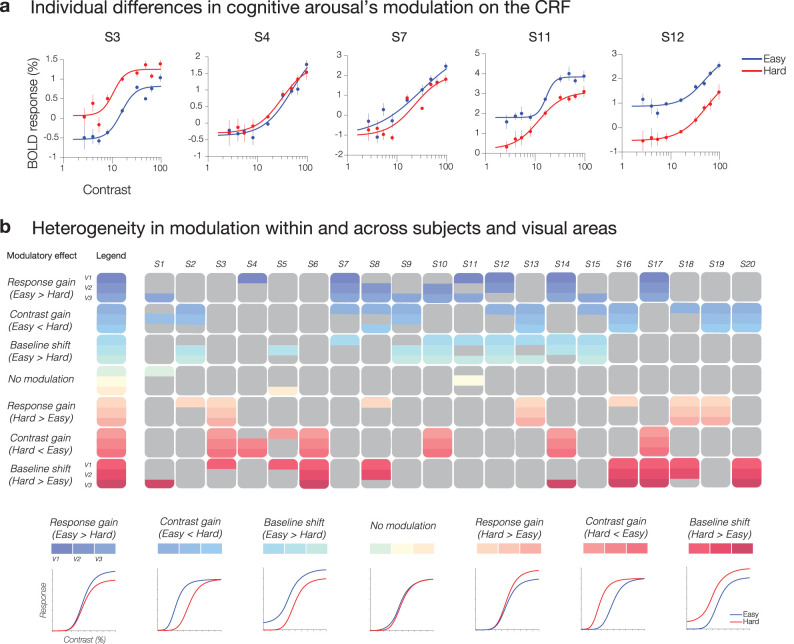
***a***, Exemplar subjects’ contrast response functions (CRFs) from visual area V3. ***b***, The modulatory patterns that best capture the modulation by cognitive arousal across subjects and visual areas V1–V3. The boxes represent the different modulations by arousal, with each color indicating a different modulatory effect. The shades and color placement within a given modulatory effect (gray box) indicate different visual areas: V1 (top color), V2 (middle color), V3 (bottom color). The colored boxes display the modulation or combinations of modulations that best capture arousal's effect within and across observers and visual areas. Across observers, there is large heterogeneity in the modulatory effect of arousal on the CRF, with groups of subjects displaying neural gain enhancements in the Easy and other individuals displaying enhancements in Hard, characterized by various combinations of response gain, contrast gain, and baseline shift patterns. Within participants, there is also variability in modulatory effects by arousal on the CRF across visual areas.

While we found no modulatory effect by cognitive arousal on the CRFs across visual areas at the group level, the absence of a modulatory effect seemed to be attributable to the diversity in modulatory responses observed across individuals (Figs. 3*C*, 4, 5). Our findings indicate that certain individuals exhibited an enhanced neural response with increased cognitive arousal, while others demonstrated the opposite effect, experiencing a decrease in neural response with heightened cognitive arousal. Figure 5 displays exemplar subjects with a wide range of distinct modulatory patterns.

How reliable were these patterns within a subject? To establish the reliability of the observed modulatory patterns within a subject, we conducted nested hypotheses tests aimed to determine whether separate fits for each arousal condition (Hard, Easy) or one combined fit for both conditions provided a better captured individual subjects’ contrast responses between the two arousal conditions (see Materials and Methods, Statistical analysis section for details). The results from these analyses confirm the general consistency of the CRFs within individuals. On average across the group, a large proportion of individual subjects’ bootstrapped CRFs yielded *F* test statistics and corresponding *p* values of <0.05 (V1 mean proportion: 65%, 95% CI [51, 79%]; V2 mean proportion: 65%, 95% CI [52, 78%]; V3 mean proportion: 64%, 95% CI [51, 77%]). This suggests that separate fits for Hard and Easy CRFs better explained the data than a single fit for both conditions, providing evidence for arousal differences on the CRF between the Easy and Hard conditions at the level of individual subjects.

### Cognitive arousal largely evokes a baseline shift in neural CRFs across voxels irrespective of modulation direction

After revealing the underlying heterogeneity in how cognitive arousal modulates the CRF across individuals, we then explored which modulatory effect best captures an individual subject's CRFs. To address this, we employed analyses at the individual voxel level for each observer and ROI to examine both the heterogeneity and consistency of modulation across voxels, within and across observers. In order to evaluate the modulation (response gain, contrast gain, baseline shift) that best characterizes cognitive arousal's impact on CRFs, we fit the data to modified versions of the Naka–Rushton function. In this modified function, we introduce an additional arousal coefficient, *A*, to explore arousal's modulation effect on the CRFs.

The response gain model equation was expressed as follows:

$$\mathrm{Response}(c)=A\;*\;(R\max-b)\frac{C^{n}}{C^{n}+C_{50}^{n}}+b,\;(2)$$

where the additional arousal parameter *A* modulates the *R*max parameter, leading to a multiplicative response gain effect, or vertical shift of the curve.

The contrast gain model equation was expressed as follows:

$$\mathrm{Response}(c)=(R\max-b)\frac{\left(A\;*\;C^{n}\right)}{(A\;*\;C^{n})+C_{50}^{n}}+b,\;(3)$$

where the additional arousal parameter *A* modulates the CRF through multiplication with the contrast intensity level, leading to a contrast gain or horizontal shift of C50 and the curve.

The baseline shift model equation was expressed as follows:

$$\mathrm{Response}(c)=(R\max-(b\;*\;A))\frac{C^{n}}{C^{n}+C_{50}^{n}}+(b\;*\;A),\;(4)$$

where the additional arousal parameter *A* modulates the *b*, or baseline parameter, representing an increase or decrease in baseline activity.

In addition to the three main models, we explored additional models by examining all possible combinations of the aforementioned three models. To determine the model that best captured the individual observer's arousal modulation on the CRF, we initially fitted the Easy (low arousal) data using the Naka–Rushton equation (Eq. 1). The resulting parameter estimates (*R*max, C50, *n*, *b*) were then treated as fixed parameters when fitting the Hard (high arousal) data with the seven models described above (e.g., Eqs. 2–4 and combination models). For each of the seven models used to fit the Hard data, the additional arousal parameter(s), *A*, was optimized in the fitting process. No upper or lower bounds were set for the arousal parameter, enabling to capture either increases (*A* > 1) or decreases (*A* < 1) in the associated fixed parameters.

To determine the most parsimonious model that explains the modulatory effect of arousal on the data, we then calculated the corrected Akaike information criterion (AIC_c_) using the sum of square errors (SSE). The corrected AIC_c_ was chosen as it better accounts for smaller sample sizes (*N* < 30; Hurvich and Tsai, 1989). To select the best and most parsimonious model, we then computed ΔAIC_c_ by subtracting the minimum AIC_c_ value among all seven models from the AIC_c_ values of each of the other models (Burnham and Anderson, 2002). A smaller ΔAIC_c_ value indicates a better fit of the model to the data compared with other models.

**Figure 6.**
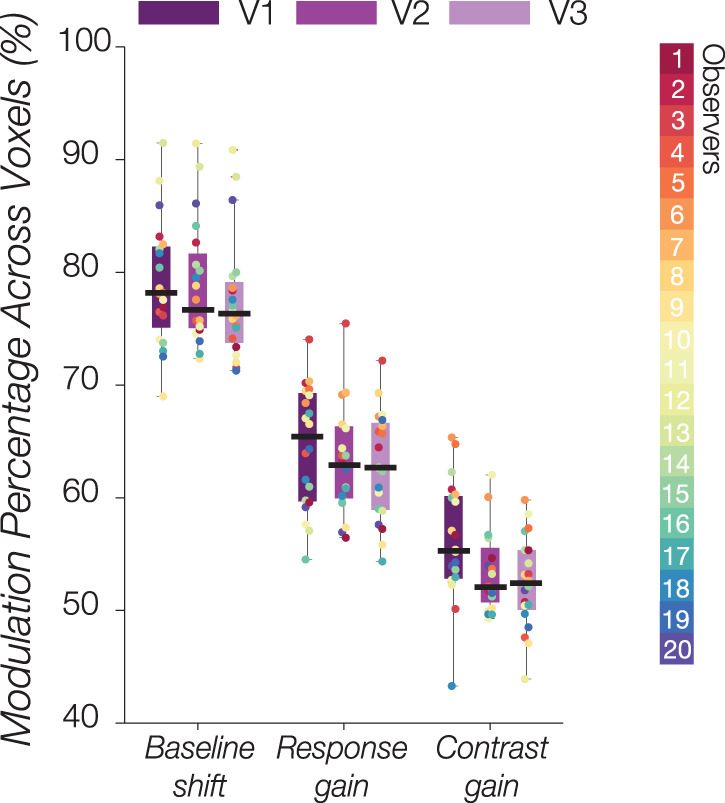
Occurrence of baseline shift, response gain, and contrast gain modulation across voxels in V1, V2, and V3. The percentage was calculated by collapsing across all combinations, irrespective of modulation direction. Overall, the most prevalent modulatory effect induced by cognitive arousal on the CRF across voxels and the entire group is the baseline shift, which remains consistent across visual areas. Each colored data point represents an individual.

In addition to substantial variability in modulation patterns across participants, there was also considerable variation in the winning model across visual areas within a participant (refer to Fig. 5*b*). However, one modulatory pattern that consistently emerged across subjects, regardless of directionality, is that of a baseline shift. When collapsing across the sign or direction of modulation (e.g., increase in baseline vs decrease in baseline with increased arousal), the top 4 modulatory patterns that captured the most voxels across subjects were the following: (1) combination of response gain and baseline shift; (2) combination of response gain, baseline shift, and contrast gain; (3) combination of baseline shift and contrast gain; and (4) baseline shift alone (Fig. 6).

To assess the overall prevalence of baseline shift, response gain, and contrast gain, we calculated the percentage of occurrence across voxels, acknowledging that they may coexist with other modulations. This analysis involved collapsing the data from the seven tested models. As expected, the majority of voxels across individuals exhibited a baseline shift modulation in response to cognitive arousal, which was consistently present across all three visual areas (V1: mean: 78.98%, 95% CI [76.38, 81.59]; V2: 78.82%, 95% CI [76.26, 81.37]; V3: 77.55%, 95% CI [74.99, 80.10]). Following this, the response gain modulation was observed (V1: 64.54%, 95% CI [61.99, 67.09]; V2: 63.29%, 95% CI [60.99, 65.58]; V3: 62.87%, 95% CI [60.62, 65.11]), followed by the contrast gain modulation (V1: 56.16%, 95% CI [53.69, 58.63]; V2: 53.32%, 95% CI [51.67, 54.96]; V3: 52.45%, 95% CI [50.54, 54.36]). Overall, a baseline shift seemed to best capture cognitive arousal's modulation via arithmetic difficulty on population CRFs in V1–V3; however, it is crucial to note that while a large proportion of voxels exhibited a baseline shift modulation, this was irrespective of sign, meaning that the baseline shift could occur in either direction, that is, a larger baseline in the Easy condition or a larger baseline in the Hard condition.

### Pupil size tracks with overall BOLD activity

One potential explanation for the observed differences in BOLD activity, particularly in relation to the directionality of the modulation on the CRFs, could relate to variation in the subjective difficulty of the arithmetic problems among participants. Such variation could have resulted in imperfect experimental control over the difference in arousal levels between the two conditions. For instance, some participants might have found the Hard condition challenging but manageable, resulting in a state of heightened alertness. On the other hand, other participants might have found the Hard condition overwhelming, causing instead, a more extreme state of arousal. To explore this possibility, we examined the potential impact of differences in pupil size, task performance (accuracy), and reaction time across subjects on the observed variations in the modulatory effect of arousal on the CRFs.

We investigated pupil size as a potential indicator of individual differences in arousal levels, as pupillometry is a well-established method for assessing arousal and effort changes in both animals and humans (McGinley et al., 2015; Vinck et al., 2015; Reimer et al., 2016; Mathôt, 2018). In the context of our experiment, differences in arousal between the two difficulty conditions could potentially be tracked or indicated by variations in pupil size. Regarding the baseline (*b*) parameter, we did not find a correlation between the difference in pupil size and baseline in visual areas V1 (*r* = −0.12, *p* = 0.62), V2 (*r* = −0.44, *p* = 0.06), or V3 (*r* = −0.48, *p* = 0.035), after correcting for multiple comparisons using Bonferroni’s correction to the number of ROIs. Furthermore, our findings revealed no significant correlation between the difference in pupil size for the Hard and Easy conditions and the parameters C50 (V1: *r* = 0.16, *p* = 0.52; V2: *r* = −0.07, *p* = 0.76; V3: *r* = −0.15, *p* = 0.53) and *R*max (V1: *r* = −0.38, *p* = 0.11; V2: *r* = −0.22, *p* = 0.36; V3: *r* = −0.15, *p* = 0.55) across all visual areas.

**Figure 7.**
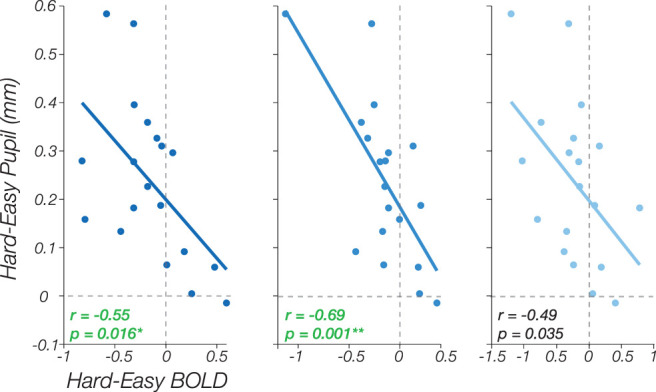
Pupil size and visuocortical response correlations (*n* = 19). Correlation between Hard-minus-Easy pupil size and Hard-minus-Easy overall BOLD response, indicative of the overall direction of modulation by arousal (i.e., increasing cognitive arousal enhances neural response or decreases neural response). Overall, there is a correlation between pupil size and overall BOLD response in V1 and V2.

However, correlations were found between the difference in pupil size and difference in overall BOLD response across all visual areas (V1: *r* = −0.55, *p* = 0.016; V2: *r* = −0.69, *p* = 0.001; V3: *r* = −0.49, *p* = 0.035; Fig. 7). Only visual areas V1 and V2 exhibit statistically significant results after correcting for multiple comparisons using Bonferroni’s corrections to the number of ROIs. Subjects who displayed a greater difference in arousal, as measured by pupil size, between the Hard and Easy conditions tended to exhibit a decrease in overall BOLD activity in the Hard condition compared with the Easy condition, and subjects who displayed a smaller difference in arousal tended to show the opposite effect—an increase in BOLD activity in the Hard condition compared with the Easy condition. This indicates that variations in pupillary size, which may be associated with variability in arousal and/or cognitive effort levels for the two difficulty conditions among participants, may be linked to the observed heterogeneity in arousal's modulation on the CRF.

Subsequently, differences in task performance (accuracy) for the Hard and Easy arithmetic problems could serve as an indirect measure of the difficulty experienced by subjects in the two conditions. On average, participants exhibited consistently high performance in the Easy task (Easy mean, 98.25%; SEM: 0.69; Accuracy range, 86.42–100%); however, across participants there was much higher variability in performance for the Hard task (Hard mean, 81.85%; SEM: 3.41; accuracy range, 35.80–99.07%). This suggests that the task difficulty for the Hard condition might have varied across individuals, with some individuals finding it more difficult than others. We examined the relationship between the difference in task performance (accuracy in %) between the Hard and Easy conditions and the corresponding signed differences in Naka–Rushton parameters (*R*max, C50, *b*), each of which capture the different cognitive arousal modulatory patterns that may impact the CRF. Moreover, we also investigated whether performance played a role in overall BOLD activity and the direction of modulation (i.e., increased neural response in Easy condition vs increased response in Hard) across individuals. Our analysis revealed no correlation between performance and the modulatory pattern of the CRF (Naka–Rushton parameters, *R*max, C50, *b*), nor the direction of modulation (overall BOLD activity) influenced by cognitive arousal across all visual areas (Table 2). Furthermore, we found no evidence of a speed–accuracy tradeoff; subjects with longer reaction times did not necessarily exhibit higher performance. No correlation was also found between reaction time and performance (*r* = −0.40, *p* = 0.08), nor between reaction time and overall BOLD activity and Naka–Rushton parameters (Table 3).

**Table 2.** Correlations between performance (accuracy) and Naka–Rushton parameter estimates and overall BOLD

ROI		*R*max	C50	Baseline	Overall BOLD
V1	Correlation with performance	*r* = 0.41	*r* = 0.23	*r* = 0.34	*r* = 0.39
	*p* value	*p* = 0.07	*p* = 0.32	*p* = 0.14	*p* = 0.09
V2	Correlation with performance	*r* = 0.26	*r* = −0.30	*r* = 0.37	*r* = 0.27
	*p* value	*p* = 0.26	*p* = 0.19	*p* = 0.11	*p* = 0.25
V3	Correlation with performance	*r* = 0.33	*r* = −0.17	*r* = 0.17	*r* = 0.12
	*p* value	*p* = 0.15	*p* = 0.47	*p* = 0.46	*p* = 0.61

**Table 3.** Correlations between reaction time and Naka–Rushton parameter estimates and overall BOLD

ROI		*R*max	C50	Baseline	Overall BOLD
V1	Correlation with reaction time	*r* = 0.22	*r* = −0.29	*r* = −0.28	*r* = 0.14
	*p* value	*p* = 0.35	*p* = 0.22	*p* = 0.24	*p* = 0.55
V2	Correlation with reaction time	*r* = 0.33	*r* = −0.19	*r* = −0.41	*r* = −0.31
	*p* value	*p* = 0.16	*p* = 0.42	*p* = 0.07	*p* = 0.18
V3	Correlation with reaction time	*r* = −0.08	*r* = −0.22	*r* = −0.24	*r* = −0.08
	*p* value	*p* = 0.74	*p* = 0.34	*p* = 0.31	*p* = 0.78

### Cognitive arousal task induces activation in task-positive regions

The observed differences in visuocortical CRF responses by cognitive arousal might also be related to variability in effort or attention exerted by participants between the two conditions. As additional analysis, we examined two network regions of interest: the DMN and the DAN. The DMN comprises three major subdivisions: the ventral medial prefrontal cortex, the dorsal medial prefrontal cortex, and the posterior cingulate cortex along with the adjacent precuneus and lateral parietal cortex. During externally oriented task performance, the DMN typically exhibits decreased activity compared with periods of relaxed nontask-related activity, especially if the task is attentionally demanding and/or goal-directed (Raichle, 2015). On the other hand, the DAN is a task-positive network, consisting of bilateral intraparietal sulcus (IPS) and frontal eye fields (FEF), and it becomes active during attentional tasks (Vossel et al., 2014).

**Figure 8.**
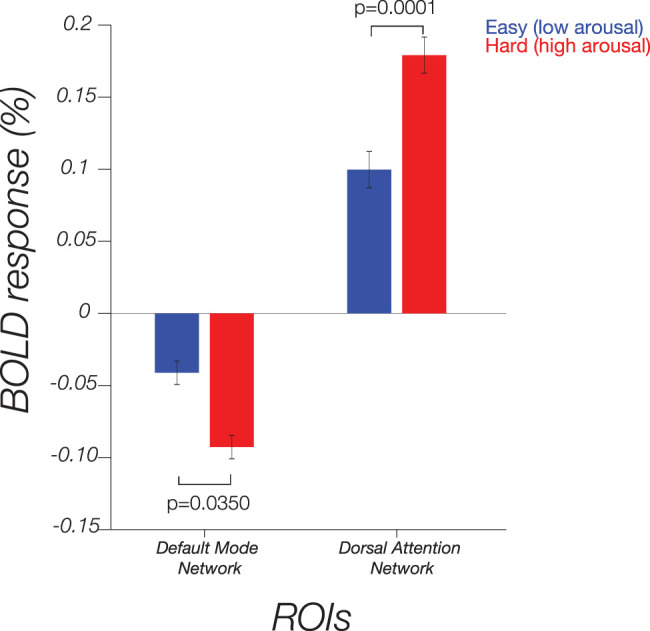
Average BOLD response in the default mode network and dorsal attention network ROIs under the Easy and Hard condition.

Consistent with previous research, we observed greater BOLD deactivation in the DMN in the Hard condition (Hard mean, −0.09; SEM: 0.03) compared with the Easy condition (Easy mean, −0.04; SEM: 0.02; *t*_(19)_ = 2.27, 95% CI = [0.004, 0.10], *p* = 0.035; Fig. 8). Regarding the DAN, we found task-positive activation, with greater BOLD activation in the Hard condition (Hard mean, 0.18; SEM: 0.03) compared with the Easy condition (Easy mean, 0.10; SEM: 0.03; *t*_(19)_ = −4.8721, 95% CI = [−0.11, −0.05], *p* = 0.0001; Fig. 8). However, only the DAN was significant after correcting for multiple comparisons, supporting the notion that the Easy and Hard conditions successfully manipulated cognitive load and/or effort (Weber et al., 2022).

## Discussion

Our vision is influenced by a variety of processes at any given moment. However, our understanding of how arousal state influences visual processing in humans remains poorly understood, which is surprising given the dynamic nature of arousal in everyday experience. In this study, we focused on exploring the influence of cognitive arousal, manipulated using arithmetic task difficulty, on population CRFs in the early visual cortex. We found that arousal's modulatory effects on visual processing can manifest in different patterns among individuals, with some experiencing enhancement and others exhibiting a decrease in visual responses. This variation might arise due to the arousal manipulation (i.e., arithmetic difficulty) employed in our study, which interacts with multiple higher-order cognitive systems and processes, whereas previous studies focused on more primary forms of arousal, such as locomotion, emotion, reward, and pain/fear (Cano et al., 2006; Phelps et al., 2006; Niell and Stryker, 2010; Zhuang et al., 2014; McGinley et al., 2015; Vinck et al., 2015; Kim et al., 2017; Shimaoka et al., 2018).

In our experiment, while we observed differences in pupil size, performance, and DAN activity between the two arithmetic difficulty conditions, we did not precisely control and titrate the difficulty levels of arithmetic problems for each individual. Consequently, this lack of titration could lead to inconsistent arousal states among individuals in the high arousal condition. For instance, one observer might experience the hard-arithmetic problems as extremely difficult and stressful, while another might find them challenging yet engaging. Interestingly, correlations between pupil size, a measure of arousal, and BOLD activity revealed that subjects with smaller differences in pupil size exhibited increased BOLD activity with cognitive arousal, whereas subjects with larger arousal differences between the two conditions tended to exhibit a decrease overall BOLD activity with cognitive arousal. Taking into consideration the potential disparity in arousal states across individuals between the two conditions, our results suggest that cognitive arousal's influence on visual contrast processing is not monotonic.

In a recent study, Sawetsuttipan et al. (2023) discovered that perceptual difficulty had a nonlinear inverted U-shaped impact on the response gain modulation of neural CRFs, analogous to the Yerkes–Dodson law, which maps the relationship between arousal and performance. This law suggests that intermediate levels of arousal lead to optimal performance, while either too much or too little can be detrimental to performance (Yerkes and Dodson, 1908; Broadhurst, 1957; Alhola and Polo-Kantola, 2007; Diamond et al., 2007). Sawetsuttipan et al.'s findings indicated that intermediate difficulty levels resulted in larger neural gain of the CRFs compared with lower or higher difficulty levels. Similar relationships have also been observed in animal studies involving rats, where the LC-NE system and sensory evoked neural response of rat thalamic neurons also followed an inverted U pattern. In this context, increasing LC-NE output, which is associated with different waking behavioral states (Aston-Jones and Cohen, 2005; Sara and Bouret, 2012), led to a peak in sensory evoked response in the somatosensory terminal fields at intermediate levels of LC-NE output and arousal but then led to a decline in evoked response with further increasing levels of LC-NE output corresponding to states of hyperarousal (Devilbiss and Waterhouse, 2000, 2004, 2011; Devilbiss et al., 2006; Waterhouse and Navarra, 2019).

**Figure 9.**
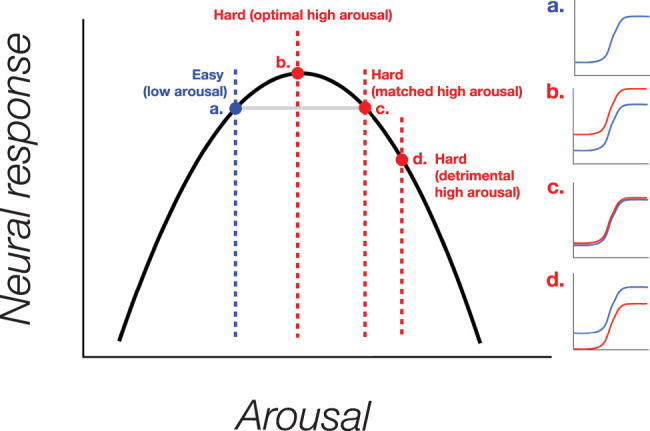
Illustration of the potential inverted U relationship between cognitive arousal and corresponding neural response of contrast response functions (CRFs), as observed in the study. Subjects displaying minimal arousal difference between difficulty conditions (Easy, Hard), as measured by pupil size, show enhanced neural responses with increasing arousal, which may be due arousal levels falling along the peak of the curve. However, as the arousal difference between the two conditions increases, a shift in the modulatory pattern occurs, resulting in a decrease in neural response for individuals with the largest pupillary difference, which could be a result of arousal levels exceeding the peak of the curve, leading to diminishing responses. It is important to note that this relationship assumes all subjects start at the same level of arousal in the Easy condition.

Therefore, in this study, the relationship between cognitive arousal and visuocortical response might also be interpreted in terms of an inverted U relationship. The polarity with which cognitive arousal modulates visuocortical responses could be linked to individual differences in the task-induced increment in arousal in the context of an underlying non-monotonic function. To illustrate this, we first assume that all participants start at the same level of arousal in the “Easy” arithmetic condition, as there was little variance around the performance level in this condition. In this case, a hypothetical starting point for an individual's arousal state in the “Easy” condition might be slightly to the left of the peak of the curve (Fig. 9, point a). Participants with the smallest pupil difference between conditions may have their arousal level in the high arousal “Hard” arithmetic condition aligned with the peak of arousal on the curve, resulting in enhanced BOLD response in the “Hard” condition (Fig. 9, point b). As the pupil size difference between conditions increases, individuals that displayed mid-level differences in pupil size across the group tended to show little-to-no difference in BOLD activity between the “Hard” and “Easy” conditions. For these participants, the arousal level in the “Hard” condition might mirror the starting point arousal level in the “Easy” condition on the other side of the peak. Consequently, even though arousal levels differ, the BOLD response to these two arousal states may exhibit similar visual responsivity (Fig. 9, point c). Lastly, individuals who exhibited the largest difference in pupil size between the two conditions may have the arousal level in the “Hard” condition fall further along the function, where increased arousal becomes detrimental to visuocortical processing, leading to a decrease in neural response in the “Hard” condition compared with the “Easy” condition (Fig. 9, point d).

While the relationship between cognitive arousal and visuocortical CRF responses presented in the illustration offers a simplified potential explanation, we acknowledge several assumptions and limitations related to using pupil size as the sole measure of arousal. Firstly, the design of this experiment does not allow for precise determination of the subjects’ arousal levels in the two conditions and direct comparisons between individuals; at most, we can only compare arousal levels within an individual. For instance, one subject may perceive the “Easy” condition as too simple, leading to disengagement or drowsiness during the task, while another subject might find the “Easy” condition engaging and stimulating. Since identifying absolute arousal levels was not the primary objective of this study, we cannot make such interindividual comparisons. It is also unclear whether similar increases in arousal consistently translate into comparable increases in both pupil and neural responses across individuals. Furthermore, although pupillometry is a popular and widely used method to measure and track arousal, in our experiment, changes in pupil size cannot solely be attributed to changes in arousal level. Pupil size changes also reflect differences in cognitive effort, load, attention, and other endogenous factors, which may interact and contribute to the arousal states observed during the arithmetic task. Distinguishing these different factors within the observed pupillary changes is challenging, as our arithmetic task likely impacts not only arousal but also other interrelated elements such as effort and attention. Therefore, future research can explore individualized arithmetic difficulty levels to accurately determine each participant's position on the Yerkes–Dodson curve under various arithmetic conditions, tasks, and arousal states.

While our arousal manipulation was chosen based on pupillometry work demonstrating the reliable impact of arithmetic difficulty on pupil size (Hess and Polt, 1964; Bradshaw, 1967; Ahern and Beatty, 1979; Steinhauer et al., 2000; Klingner et al., 2011; Pan et al., 2022), a limitation of this manipulation is the inability to disentangle the differential roles of arousal, attention, and higher-order cognitive processing (e.g., effort, load) in modulating both pupil size and visuocortical responses. Furthermore, the use of auditory stimuli, which have been shown to influence visuocortical responses (Cate et al., 2009; Petro et al., 2017), introduces the possibility of a potential interaction between the auditory stimuli and the observed visual responses. Moreover, observers likely allocated varying levels of attention toward the auditory stimuli in the Hard versus Easy conditions, with these attentional differences potentially interacting or intertwining with arousal levels. The arithmetic manipulation could be influenced by various factors, including differences in problem-solving strategies, expertise, expended effort, psychological stress from arithmetic, and more (LeFevre et al., 1996; Campbell and Xue, 2001; Lemaire and Lecacheur, 2010). Future research can delve into the contributions of these different processes and their interactions in influencing pupillary size and visual processing. Additionally, exploring more primary forms of arousal, such as endogenous fluctuations, locomotion, pain, and emotion, in future studies could provide insight into whether arousal's modulation in these less cognitive-driven arousal states yields more consistent effects on visuocortical responses across individuals or whether individual differences still persist. Interestingly, previous studies examining the effects of various forms of arousal, such as emotion, aversive stimuli, rewards, stress, and anxiety, on early visual processing have yielded mixed results across studies, with some showing improvement and others indicating impairment in performance [e.g., improvement (Phelps et al., 2006); impairment (Most et al., 2007); combination (Song and Keil, 2013)], suggesting that the Yerkes–Dodson law may also play a role in various forms of arousal. Further work is needed to parametrically manipulate the level of arousal to map out individual subjects’ Yerkes–Dodson functions of arousal and to test whether arousal and cognitive processes exhibit an inverted U relationship with sensory processing.

Another limitation of our study is the potential influence of task-related effects on the observed visual effects. In our paradigm, the presentation of visual stimuli (contrast gratings) is always concurrent with the auditory arithmetic tasks, making it difficult to disentangle the effects tied to the task itself from those specifically related to the visual stimulation. Our results do not establish that the task-related effects depend on an interaction with vision or would go away without a visual stimulus. Previous research has demonstrated that task-related responses, modulated by factors such as task difficulty and arousal, can significantly influence fMRI activity in early visual cortex independently of visual stimulation (Cardoso et al., 2019; Roth et al., 2020; Burlingham et al., 2022). For instance, Burlingham et al. (2022) observed increased response amplitudes in early visual cortex during more difficult conditions and in trials where participants made errors, likely reflecting an arousal-related effect. Similarly, studies examining the influence of reward on task-related responses have reported larger response amplitudes and lower trial-to-trial variability with higher rewards compared with lower rewards (Cardoso et al., 2019; Roth et al., 2020). Therefore, there is a possibility that the modulatory effects on visual cortex observed in our study are not solely due to task or arousal effects but rather reflect an interaction between task-related responses and visual responses. While our current paradigm does not allow for a separation of these influences, future investigations could aim to adopt a paradigm that separates the task from the visual responses to better investigate the individual contributions of each to visual processing. Doing so could also enable more nuanced investigations of the effects of stimulus and task-related components on pupil size (Kim et al., 2023) and its role and relationship with neural processing.

Taken together, our study reports individual differences in cognitive arousal's modulation of visual responses, with some individuals exhibiting enhanced neural responses with arousal, while others display a decrease, and other subjects scattered in between. Further research is essential to fully understand this relationship and uncover the underlying mechanisms and potential interactions with higher-order cognitive areas or different processes that may account for the diverse effects observed.
